# Bridging the gap in paediatric MASLD screening: progress, pitfalls and the path ahead

**DOI:** 10.1038/s41390-026-04763-5

**Published:** 2026-02-24

**Authors:** Sunitha Vimalesvaran, Anil Dhawan

**Affiliations:** 1 Department of Paediatric Gastroenterology, Hepatology and Nutrition, National University Hospital, Singapore, Singapore; 2 Paediatric Liver, GI and Nutrition Center and Mowat Labs, King’s College Hospital, London, UK

## Abstract

With a significant rise in the number of children with obesity worldwide, there has been a parallel increase in metabolic dysfunction-associated steatotic liver disease (MASLD), now the most common chronic liver disease in youth. MASLD remains substantially underdiagnosed, largely due to its asymptomatic early course, variability in guideline-recommended screening thresholds, and limited access to paediatric-specific diagnostic tools. In a large real-world cohort of 908 overweight and obese children, Lehtinen et al. provide one of the most comprehensive evaluations of MASLD screening implementation in routine care. Whilst screening uptake for ALT and metabolic comorbidities was high and broadly in line with international guidelines, under-recognition persisted, with retrospective MASLD prevalence nearly double clinician-documented rates. The study highlights limitations of ALT-based screening and inconsistent diagnostic criteria. It also reinforces the importance of structured exclusion testing to avoid misclassification of inherited or treatable liver diseases as MASLD. Current non-invasive tools for steatosis and fibrosis remain inadequately validated in children, underscoring an urgent unmet need in paediatric hepatology. Together, these findings emphasise the necessity for harmonised screening pathways, improved diagnostic algorithms, and broader access to practical non-invasive tools to enable earlier MASLD detection and intervention.

With more than 340 million children and adolescents affected globally, childhood obesity has become one of the most pressing epidemics of our time.^1^ The implications are profound; conditions such as cardiovascular disease, type 2 diabetes (T2DM), and even cancer - once rarely seen in the young, are now appearing with unsettling frequency. Reflecting this broader shift, MASLD has rapidly become the most common chronic liver disease in children worldwide - estimated to affect around 13% of individuals aged 18 years and younger.^2–4^ Yet, it remains substantially underdiagnosed. The majority of these children remain untreated, increasing the likelihood of progression into adulthood.^5^ This largely silent trajectory highlights MASLD as a major contributor to chronic liver disease in youth and an escalating public health challenge.

Early detection and treatment of MASLD as a comorbidity of obesity is cornerstone in the prevention of further metabolic disease such as T2DM,^6^ but screening, especially in children, remains inherently challenging. The disease is often asymptomatic in its early stages, allowing significant hepatic injury to accumulate before clinical detection is possible.^7^ Guidance across major societies such as ESPGHAN, NASPGHAN, and national bodies also varies in recommended age thresholds, screening tools, and ALT cut-offs, leading to inconsistent practices in real-world settings.^8,9^ Moreover, effective screening requires resources such as laboratory capacity, access to paediatric ultrasonography, and trained clinicians - elements that are not uniformly available, particularly in primary care or lower-resourced health systems.^10^ These factors collectively contribute to delayed diagnosis and the persistent under-recognition of MASLD in paediatric populations.

The study by Lehtinen et al.^11^ constitutes a rigorous real-world evaluation of paediatric MASLD screening practices within a public healthcare setting, addressing a notable evidence gap in the implementation of guideline-based recommendations. Drawing on a large cohort of 908 overweight or obese children, the authors demonstrate that screening uptake, particularly ALT testing (83.1%) and assessment of metabolic comorbidities (87.9%), is generally high and largely concordant with established guidelines. Nonetheless, the study also reveals important deficiencies, including substantial underdiagnosis despite frequent testing (7.4% clinical vs. 14.5% retrospective), inconsistent use of ultrasonography and differential diagnostic workups, and the observation that a considerable proportion of younger children, below recommended screening ages, were both screened and found to meet MASLD criteria. Conducted across nearly two decades, this work provides valuable insight into how screening recommendations are operationalised in clinical practice and identifies areas where implementation may be optimised.

These findings are based on a well-defined cohort of children from both primary and tertiary care, enabling a comprehensive assessment of real-world screening practices across the healthcare continuum. Population-based electronic health records enhances data completeness and reduces selection bias although the use of obesity-related ICD codes may not be the most accurate at identifying cases. The longitudinal nature of the study over two decades allows for meaningful analysis of secular trends. The application of current MASLD criteria retrospectively, provides valuable insight into the underdiagnosis of the condition and impact of evolving definitions. Additionally, evolving guidelines and practice patterns introduces heterogeneity in screening thresholds across this period of time. Because the risk of MASLD varies significantly across racial and ethnic groups,^12^ the homogenous ethnicity of this study restricts generalisability to more diverse settings.

This study underscores some of the pivotal progress made in paediatric MASLD care but also the persistent gaps that exist in detection within routine care. The shift from NAFLD to MASLD inevitably raises questions about the applicability of earlier research conducted under previous nomenclature.^13^ However, accumulating evidence suggests that substantial concordance exists across these definitions, particularly in obesity-related populations, supporting cautious extrapolation of prior findings to MASLD. In US paediatric cohorts, approximately 80% of children with NAFLD also met MASLD criteria, while in a Chinese paediatric obesity population this overlap was 100%, indicating near-total alignment in children with excess adiposity.^14,15^ Therefore, whilst terminological changes reflect evolving pathophysiological understanding, the populations captured under NAFLD, MAFLD, and MASLD are highly overlapping, particularly in paediatric obesity, rendering prior evidence broadly informative for current MASLD-focused analyses.

This study also begs the question of what the most appropriate “screening tool” is for MASLD in children. Despite high rates of ALT testing and comorbidity screening, the nearly twofold discrepancy between clinician-diagnosed cases and those meeting MASLD criteria retrospectively highlights ongoing under-recognition, likely driven by the limited sensitivity of ALT alone (mediocre sensitivity, indirect marker of steatosis and within-individual variability). Additionally, substantial variation in recommended thresholds across guidelines exists. Proposed cut-offs range widely; from 22 U/L for girls and 25 U/L for boys (Endocrine Society),^16^ to 35 U/L (ESPGHAN),^17^ and 44–52 U/L depending on sex (NASPGHAN/AAP)^18^; while some guidelines do not specify a threshold at all. In addition to these discrepancies, recommendations for subsequent management after a positive screen also differs, contributing to inconsistent clinical practice and uncertainty regarding optimal diagnostic pathways. However, ALT testing is cheap, easily available and practical compared to other currently available modalities.^19^ It underscores the need for more harmonised ALT cut-off values and improved diagnostic pathways for MASLD in children.

An additional and often underappreciated challenge in paediatric MASLD screening is the risk of misclassifying children with underlying inherited or treatable liver diseases as having MASLD solely on the basis of elevated ALT or the presence of obesity. Given the high prevalence of overweight and obesity in childhood, conditions such as Wilson disease, autoimmune hepatitis, alpha-1 antitrypsin deficiency, and other metabolic or genetic hepatopathies may be inadvertently overlooked unless a structured exclusion process is undertaken. Hegarty et al. demonstrated that a significant proportion of paediatric fatty liver presentations arise from non-MASLD etiologies, underscoring the necessity of a systematic diagnostic framework before attributing liver abnormalities to MASLD.^20^ This reinforces the argument for an integrated screening strategy that couples ALT-based screening with age-appropriate metabolic risk assessment and a consistent set of exclusion tests, particularly for younger children who may otherwise be dismissed as having obesity-related liver disease.

Younger children were also screened and found to have abnormalities, aligning with emerging evidence that MASLD can present earlier and may be more aggressive in youth.^21^ Unlike in adults, non-invasive tools for detecting steatosis and fibrosis in children are far less validated and substantially less reliable. Common adult biomarkers and prediction scores such as the Fatty Liver Index, NAFLD Liver Fat Score, APRI, and FIB-4; demonstrate poor accuracy or require further validation in paediatric populations.^22^ Similarly, although ultrasound is widely available, it has limited sensitivity for mild steatosis (<30%) and is unreliable for monitoring longitudinal changes.^23^ More advanced modalities such as controlled attenuation parameter (CAP) and MRI-PDFF offer improved accuracy for quantifying hepatic fat, but CAP thresholds for children remain undefined and MRI is constrained by cost, limited availability, and the need for sedation in younger age groups. Tools for fibrosis assessment, including transient elastography (FibroScan) and magnetic resonance elastography, also lack robust paediatric performance data.^24^ Eventually, community- or school-based access to simple non-invasive tools, such as point-of-care FibroScan, could serve as a valuable adjunct, enabling early identification of children with hepatic fibrosis/steatotic changes while prompting timely referral for further metabolic or autoimmune workup.

Collectively, these limitations underscore a critical gap: the absence of a validated, practical, and accurate non-invasive test for MASLD in children. This gap is especially consequential for younger children, in whom early disease may be missed. These constraints reinforce the need for an integrated screening strategy that combines ALT interpretation, metabolic risk profiling, and selective use of non-invasive imaging to improve early MASLD identification in this vulnerable population. Strengthening such frameworks will be essential to facilitate earlier identification and timely intervention, ultimately mitigating long-term progression to advanced liver disease.
